# Dislocation of the Astragalus and Subsequent Extraction of the Bone—Foot Preserved

**Published:** 1832

**Authors:** Thomas Wells

**Affiliations:** Columbia, South Carolina


27. *Dislocation of the Astragalus and subsequent Extraction of the bone—Foot preserved.* By Thomas Wells, M. D., of Columbia, South Carolina.—Dr. G. W. L. taking a ride during his convalescence from an attack of bilious fever, dislocated the astragalus from its union with the os scaphoides, by imprudently leaping from his gig. Violent efforts were made to reduce the bone, which were followed by inflammation and ulceration, so as to expose the head of the astragalus, which soon after became carious.

Six months afterward he came to Columbia, his general health still impaired; the ancle swelled, occasionally painful, and admitting of little motion; the foot turned inwards and partially extended—an ulcer three-fourths of an inch in diameter exposed the carious head of the bone. He walked on crutches, and could sustain little weight on the injured foot.

Towards the latter part of July, too much exercise brought on a high degree of inflammation, with severe pain and general fever. Notwithstanding the most rigid antiphlogistic means, general and local, extensive suppuration took place, and the matter was discharged by puncture. To these symptoms succeeded hectic fever, with a copious purulent discharge, until the patient was very much reduced, the astralagus was found to be carious at several points, and its removal was decided upon as the only alternative for amputation, and was accordingly performed on the 18th of August.

An incision was made, commencing at the edge of the original ulcer, near the tendon of the common extensor of the toes, and carried a little backward and downward beyond the lower head of the fibula, and the bone carefully detached from its connexions. No vessel was divided sufficiently large to require a ligature, yet the wound presented a frightful appearance, the foot seeming to be nearly separated from the leg.

By suitable support the limb was kept in a position at right angles with the leg. He suffered little after the operation. The wound, which at first discharged copiously, granulated and healed kindly, subject only to occasional interruptions from derangement of the digestive organs, and by the end of September was perfectly healed. Twelve months after the operation Dr. W. again saw him the leg: was shortened about an inch, but the deficiency being supplied with a high heeled shoe, he walked without difficulty.—*Ib.*

